# Chronic Kidney Disease and Exposure to Nephrotoxic Metals

**DOI:** 10.3390/ijms18051039

**Published:** 2017-05-12

**Authors:** Sarah E. Orr, Christy C. Bridges

**Affiliations:** Mercer University School of Medicine, Division of Basic Medical Sciences, 1550 College St., Macon, GA 31207, USA; sarah.elizabeth.orr@live.mercer.edu

**Keywords:** chronic kidney disease, arsenic, cadmium, lead, mercury

## Abstract

Chronic kidney disease (CKD) is a common progressive disease that is typically characterized by the permanent loss of functional nephrons. As injured nephrons become sclerotic and die, the remaining healthy nephrons undergo numerous structural, molecular, and functional changes in an attempt to compensate for the loss of diseased nephrons. These compensatory changes enable the kidney to maintain fluid and solute homeostasis until approximately 75% of nephrons are lost. As CKD continues to progress, glomerular filtration rate decreases, and remaining nephrons are unable to effectively eliminate metabolic wastes and environmental toxicants from the body. This inability may enhance mortality and/or morbidity of an individual. Environmental toxicants of particular concern are arsenic, cadmium, lead, and mercury. Since these metals are present throughout the environment and exposure to one or more of these metals is unavoidable, it is important that the way in which these metals are handled by target organs in normal and disease states is understood completely.

## 1. Introduction

Chronic kidney disease (CKD) is becoming increasingly common worldwide. Recent estimates suggest that 8–16% of the global population is affected by some form of CKD [1]. As the prevalence of obesity, diabetes, and hypertension increases, the risk of developing CKD also increases. As CKD progresses, patients have a reduced ability to eliminate metabolic wastes, xenobiotics, and toxicants. Of particular concern is the inability of these patients to mediate the urinary excretion of prevalent environmental toxicants. Since the environment is heavily contaminated by metal toxicants such as arsenic, cadmium, lead, and mercury, human exposure to one or more of these toxicants is nearly unavoidable. Understanding the way in which these metals are handled by diseased kidneys addresses an important global health problem.

## 2. Chronic Kidney Disease

**Figure 1:**
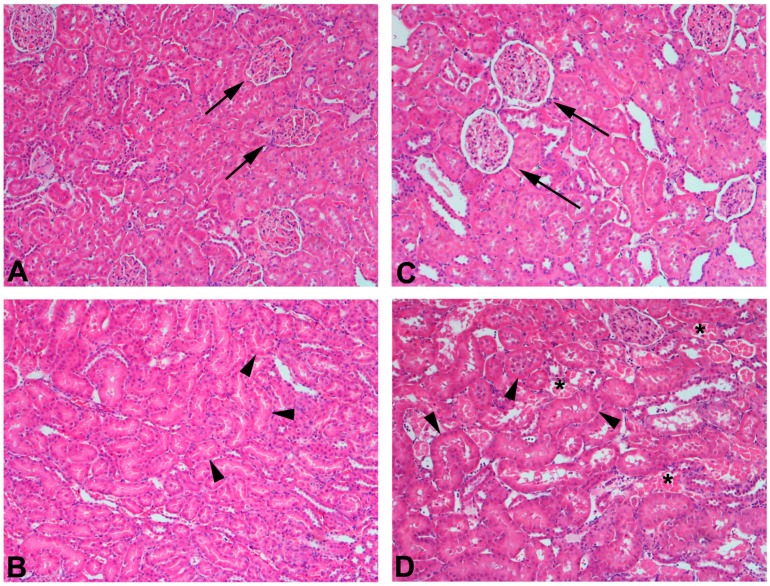
Histological sections of kidneys from Sham or 75% nephrectomized Wistar rats. Normal glomeruli (arrows) and tubules from the renal cortex are shown in panel (**A**) while panel (**B**) shows normal tubules (arrowheads) from the outer stripe of the outer medulla. Sections of renal cortex (**C**) and outer stripe of outer medulla (**D**) from a 75% nephrectomized rat are also shown. A 75% nephrectomized rat is considered to be an appropriate model of chronic kidney disease. Glomeruli (arrows (**C**)) from a 75% nephrectomized rat appear to be hypertrophied as a compensatory response to a reduction in renal mass. Similarly, the tubules in the outer stripe of the outer medulla (**D**) of the 75% nephrectomized rat also appear to be hypertrophied (arrowheads). In contrast, some tubules (*) display signs of necrosis, which is likely due to the reduced perfusion of blood to those nephrons. Magnification, 100×.

CKD is characterized by a permanent loss of nephrons and an eventual decline in glomerular filtration rate (GFR) [2]. These alterations are accompanied by structural, functional, and molecular changes in the remaining functional nephrons in an attempt to compensate for the loss of diseased nephrons. These changes include glomerular cellular and tubular hypertrophy (Figure 1), enhanced renal blood flow, and enhanced single nephron glomerular filtration rate (SNGFR) [3]. In addition, transcription and translation of RNA is enhanced which leads to increases in mRNA expression and protein levels. Together, these compensatory changes increase the delivery of solute to healthy nephrons and enhance the rate of solute uptake by tubular epithelial cells [3,4,5,6]. In general, the overall functional capacity of hypertrophied nephrons is enhanced as a mechanism to compensate for the loss of diseased/injured nephrons. 

Unfortunately, the compensatory changes in healthy nephrons can eventually lead to injury, sclerosis, and death of those nephrons. Once the population of functional nephrons is reduced to approximately 25% of normal, compensatory changes in nephrons are no longer sufficient to maintain fluid homeostasis, and proper renal function. When the total GFR is decreased, endogenous wastes, xenobiotics, and toxicants can accumulate in the blood and cause metabolic disturbances and/or organ intoxication [7]. The accumulation of these substances may enhance morbidity and/or mortality of patients affected by CKD.

## 3. Chronic Kidney Disease and Exposure to Toxic Metals

As described previously, CKD is associated with hyperperfusion and an increase in SNGFR [7,8]. Consequently, the luminal and basolateral surfaces of renal tubular epithelial cells are potentially exposed to higher levels of xenobiotics, metabolic wastes, and nephrotoxicants. In addition, these substances may be taken up more readily by hypertrophied tubular cells because of increases in the expression of certain cellular transport mechanisms [3,9]. The increased exposure to, and probable uptake of, available xenobiotics, metabolic wastes, and nephrotoxicants likely enhances the risk of hypertrophied tubular cells being affected adversely by these substances [10]. Indeed, it has also been suggested that exposure to heavy metals can negatively alter the function of the remaining functional nephrons [11,12]. These adverse effects could conceivably lead to additional and/or more rapid cell death and glomerulosclerosis, which would further reduce the functional renal mass of the patient. It is important to consider that as the functional renal mass of the patient is reduced, urinary excretion of xenobiotics and toxicants is also reduced, which may negatively affect the overall health of the patient. Indeed, in vivo studies show that exposure of uninephrectomized rats to nephrotoxicants results in cellular injury that is more extensive than that of normal, healthy rats exposed to the same nephrotoxicants [13].

Owing to naturally-occurring and chemically manufactured toxicants in the environment, individuals are exposed frequently over their lifetime to toxicants that have the capacity to negatively affect various organ systems. Since all relevant nephrotoxicants cannot be addressed here, we will focus on some of the most prevalent environmental metal toxicants: arsenic, cadmium, lead, and mercury.

## 4. Environmentally Relevant Toxic Metals

### 4.1. Arsenic

Arsenic (As) is a highly toxic metalloid found ubiquitously in the earth’s crust. It accumulates naturally in aquifers through anthropogenic activities. In the environment, it is usually bound to oxygen, chlorine, or sulfur, and is referred to as inorganic As (iAs) [14], which is recognized by the Centers for Disease Control and Prevention as a human poison and carcinogen [14]. Humans are exposed to iAs through environmental, dietary, and occupational sources. The primary route of human exposure to iAs is via the ingestion of drinking water contaminated with iAs, as trivalent arsenite (iAs^III^) and pentavalent arsenate (iAs^V^) [15,16]. Furthermore, children who play often in sand may unintentionally ingest dirt contaminated with iAs. Humans may also be exposed to iAs upon ingestion of certain fish and various types of seafood, which may contain high levels of this metal. With regard to occupational sources of iAs in the United States, approximately 90% of all iAs used in industry is utilized in the pressure-treatment of wood. Workers involved in the treatment process or exposed to dust produced when the wood is cut are at higher risk for As intoxication. 

Ingested iAs is readily absorbed in the intestinal tract [17] and can lead to nausea, vomiting, hyperkeratosis, anemia, diabetes, cardiovascular disease, and certain types of cancer [18,19,20,21]. Once ingested, arsenic (III) methyltransferase (AS3MT) mediates the *S*-adenosylmethionine (SAM)-dependent methylation of iAs to tri- and pentavalent forms of methylated arsenic [22]. AS3MT has been detected in human liver, kidney, bladder, heart, lung, testes, and adrenal gland [22,23]. In humans, the four major methylation products are methylarsenic acid (MAs^V^), methylarsonous acid (MAs^III^), dimethylarsinic acid (DMAs^V^), and dimethylarsinous acid (DMAs^III^) [16,24]. It has been suggested that the trivalent forms of As, MAs^III^ and DMAs^III^, are more toxic than the pentavalent forms, MAs^V^ and DMAs^V^ [25,26,27,28,29,30,31,32].

Human exposure to methylated forms of As (MAs and DMAs) usually occurs via exposure to pesticides used for treatment of cotton crops [14]. Interestingly, ingestion of methylated forms of As appears to have fewer toxicological effects than ingestion of the same dose of iAs [14]. Exposure to high doses of Mas and DMAs are required to produce the same toxicological effects as exposure to a lower dose of iAs. 

#### 4.1.1. Renal Handling of Arsenic

Urinary excretion represents the major route of As elimination [33]. Since a large fraction of absorbed As is filtered at the site of the kidney, the kidney is an important site of As uptake and accumulation. Studies using yeast and *Xenopus laevis* oocytes suggest that the glucose transporter, GLUT1 (*SLC2A1*), and possibly GLUT5 (*SLC2A5*), may play a role in the uptake of As^III^ and MAs^III^ at the basolateral membrane of proximal tubular cells (Table 1) [34]. These carriers are localized in the basolateral membrane of proximal tubular cells [35,36] and may mediate the uptake of As^III^ and MAs^III^ from peritubular capillaries into proximal tubular cells. Interestingly, studies in *Xenopus laevis* oocytes suggest that the uptake of As^III^ may also be mediated by aquaporin 3 (AQP3) [37]. AQP3 is localized in the basolateral membrane of renal distal tubules and collecting ducts [38] and, thus, it may play a role in the basolateral uptake of As^III^ by renal tubules. In addition, the organic anion transporting polypeptide, OATP2B1 (*SLCO2B1*), may be involved in basolateral uptake of some forms of As [39]. This carrier has been implicated in the proximal tubular uptake of cisplatin and since cisplatin and As are both heavy metals, it is possible that OATP2B1 may also play a role in the uptake of arsenicals [40]. However, there is no direct evidence suggesting that OATP2B1 is capable of mediating the transport of any form of As.

The mechanisms by which iAs^III^ and MAs^III^ are transported out of renal tubular epithelial cells are not completely clear at present. However, it has been suggested that since GLUT1 and GLUT5 are capable of bidirectional transport, they may be able to mediate the export of As^III^ and MAs^III^ [41]. Similarly, OATP2B1 has been shown to mediate bi-directional transport of certain substrates and, thus, it may also be capable of mediating the export of As^III^ and MAs^III^ [41]. The ability of these carriers to mediate the efflux of any species of As has not been shown. 

Alternatively, glutathione (GSH) conjugates of arsenicals may be transportable forms of As at the site of specific transport proteins in renal tubular epithelial cells. Indeed, studies using in vitro models suggest that the transport of As in certain cells is dependent upon the availability of GSH [42,43,44]. Like many other metals, iAs^III^ and MAs^III^ appear capable of binding to free GSH, thereby forming more transportable forms of As, (i.e., As(GS)_3_ and MAs(GS)_2_, respectively) [45]. It appears that iAs^III^ and GSH form a complex consisting of three GSH molecules bound to one As atom (As(GS)_3_) [46,47]. Similarly, one atom of MAs may bind to two GSH molecules to form MAs(GS)_2_. The resulting arsenic species may be exported into the tubular lumen via apical export proteins such as the multidrug resistance-associated protein 2 (MRP2; *ABCC2*) and/or P-glycoprotein (*MDR1*; *ABCB1*) [48]. Interestingly, exposure of mice to inorganic species of As, enhanced the mRNA expression of MRP2 [49]. Similarly, exposure of cultured hepatocytes to sodium arsenite or sodium arsenate led to a dose- and time-dependent increase in the mRNA expression of MRP2 [49]. The As-induced increase in expression of MRP2 suggests that this carrier plays an important role in the export of As ions out of the cells for eventual excretion in the urine. Additional studies in cultured hepatocytes demonstrate that As(GS)_3_ and MAs(GS)_2_ are exported out of hepatocytes into bile via MRP2 [50,51]. Since MRP2 is also found in the apical membrane of proximal tubular cells [52], it is possible that MRP2 and P-glycoprotein may also play roles in the export of arsenic species from proximal tubular cells. In vivo experiments in mice treated with MK-571, an inhibitor of MRP2, showed that inhibition of this carrier reduced the urinary excretion of As by 50% [42]. More direct studies indicate that As(GS)_3_ and MAs(GS)_2_ can be transported out of proximal tubular cells via MRP2 [37,53]. Similarly, MRP4 (*ABCC4*), which is also present on the apical plasma membrane of proximal tubular cells, appears to play a role in the export of iAs^III^, iAs^V^, MAs^III^, MAs^V^, and/or DMA^V^ [54]. 

With regard to P-glycoprotein, studies using *mdr1* knockout mice suggest that there is an additional transporter involved in the export of As complexes out of renal tubular epithelial cells [42]. In addition, in vivo studies have shown that the expression of P-glycoprotein in the kidney is upregulated following As-induced cellular injury [55,56]. Studies in *mdr1* knockout mice indicate that the accumulation of iAs is greater in liver, kidney, and small intestine of the knockout mice compared to wild type. The knockout mice were also more susceptible to intoxication by As than wild type mice [57,58]. Together, these data suggest that P-glycoprotein may be involved in the export of iAs into the tubular lumen for excretion in urine.

The metal and toxicant extrusion protein (MATE; *SLC47A1*) may also play a role in the export of As compounds at the luminal membrane of proximal tubular cells. This carrier is localized in the apical membrane of proximal tubular cells and has been shown to mediate the export of a wide variety of xenobiotics and toxicants [59]. There are no data suggesting that it may mediate the export of As compounds, however, it has been shown to mediate the export of Cd [60] and, thus, we suggest that it may also play a role in the export of As.

#### 4.1.2. Renal Effects of Arsenic Exposure

Acute arsenic-induced renal intoxication may lead to tubulointerstitial nephritis and acute tubular necrosis [61]. As-induced kidney injury is characterized by hypercalciuria, albuminuria, nephrocalcinosis, and necrosis of the renal papillae [14,62]. In vivo and in vitro studies suggest that transport of iAs into cells may lead to alterations in intracellular signaling processes. Exposure of mice to iAs reduced the expression of raf kinase inhibitor protein (RKIP) [63], which is involved in the regulation of multiple cell signaling cascades [64,65,66]. RKIP is thought to play an important role in the regulation of growth and the survival of cells, suggesting that downregulation of its expression may lead to uncontrolled growth of cells and cancer [66]. In addition, exposure to As compounds appears to lead to DNA methylation and histone acetylation, which, in turn, may cause epigenetic alterations in the expression of interleukin-8 (IL-8) in renal tubular cells [67]. These alterations have been shown to result in proneoplastic changes such as increased migration, proliferation, and cell-cycle dysregulation in renal cells [67]. These types of alterations may represent early steps in the progression of certain types of cancer.

Cellular injury and death following exposure to iAs is likely related to the As-induced generation of reactive oxygen species (ROS) [68]. Indeed, oxidative stress has been shown to be a mechanism of iAs-induced cellular intoxication [69]. Oral exposure of rats to or direct treatment of cells with sodium arsenite has been shown to increase production of ROS and reactive nitrogen species (RNS); enhance lipid peroxidation, protein carbonylation, DNA oxidation; and reduce antioxidant defenses [70,71,72]. Similarly, exposure of cultured renal epithelial cells (HEK-293) to iAs led to elevated ROS and enhanced the expression of heme oxygenase (HMOX1), which regulates heme oxidation and stress responses [73]. Mitochondria appear to be important targets of As-induced cellular toxicity in that they are highly sensitive to oxidative injury [74,75]. 

Interestingly, in vivo and in vitro experiments have shown that exposure to arsenicals enhances the expression of metallothionein (MT) in a dose-dependent manner [76,77,78]. Computational modeling suggests that As binds to free thiols on the MT molecule [79,80], which may reduce the oxidative injury induced by exposure to As [81]. Interestingly, it has been suggested that the formation of MT-As complexes does not occur following acute exposure to iAs; rather, it was postulated that these complexes are formed only in cases of chronic exposure [82]. 

#### 4.1.3. CKD and the Effects of Arsenic Exposure on the Kidneys

Environmental, occupational, and dietary exposure to As appears to contribute to the incidence of renal injury and the development of renal disease. Contamination of drinking water with As has been linked to the development of hypertension and renal injury [15,83]. Similarly, a review of 24 case studies suggests that there is a positive association between exposure to iAs and kidney injury. Exposure to iAs was shown to cause albuminuria and proteinuria but it did not enhance other outcomes (e.g., anemia, hyperkalemia, hypocalcemia) that are associated with CKD. Therefore, the authors concluded that although exposure to iAs appears to cause renal injury, there is not a clear association between iAs exposure and the development of CKD [22]. In contrast, findings from a cross-sectional study of patients in Taiwan showed a positive correlation between urinary levels of As and the incidence of CKD. It was concluded that high levels of urinary As may increase the risk of developing CKD by four-fold [84]. The increased risk of CKD as a consequence of As exposure may be due to nephron death and compensatory changes in healthy nephrons that lead to a progressive loss of nephrons. In support of this idea, results from the National Health and Nutrition Examination Survey 2009–2012 indicate that early, acute exposure to As may lead to increased GFR [83], which suggests that as nephrons are injured, the remaining healthy nephrons compensate by increasing SNGFR and ultimately, total GFR [85]. When compensatory changes lead to injury and death of healthy nephrons, patients may develop CKD. When CKD develops, the urinary excretion of As and other toxicants has been shown to decrease, which may lead to additional toxicological effects in target cells and organs [86]. 

### 4.2. Cadmium

Cadmium (Cd) is a prevalent environmental pollutant and nephrotoxicant. Industrial uses of Cd include manufacture of batteries, pigments, coatings, and plastics [87]. Current regulations regarding Cd emissions and disposal have reduced occupational exposure to Cd, yet the environment surrounding areas where Cd is/was used industrially remain heavily contaminated. Additionally, the use of this metal in phosphate fertilizers can leave soil and water contaminated heavily with Cd residue. Cd concentrates in soils and subsequently accumulates in plants, particularly root vegetables, grains, and tobacco [88]. Cd is also present in high concentrations in aquatic animals, such as seals and mollusks, and in crustaceans, such as oysters and crabs [88]. 

Diet is the primary means by which the general, non-smoking population is exposed to Cd [88]. In contrast, because of the high concentration of Cd in tobacco, individuals who smoke tobacco products are exposed regularly to this metal [88]. Indeed, most cigarettes contain approximately 1–2 µg of Cd [89]. About 10% of the Cd contained in a cigarette is inhaled [90], with approximately 50% of that being absorbed in the lungs [91]. Therefore, it is estimated that individuals who smoke one pack of 20 cigarettes each day will absorb approximately 1–2 µg of Cd daily [92]. Cd is also present in air and drinking water in various regions of the world, although the concentration of Cd in air is relatively low and drinking water is normally not a major source of exposure for the general population [88,92,93].

In a recent assessment by the US Centers for Disease Control and Prevention, as part of the National Health and Nutrition Examination Study (NHANES), blood and urine of over 5000 individuals was analyzed for Cd. In individuals over the age of 20, the average blood level was 0.376 µg/L while the average urinary concentration of Cd was 0.232 µg/L [94]. Both urinary and blood levels of Cd have remained fairly steady over the past decade. These data suggest that individuals continue to be exposed chronically to Cd. Thus, a thorough understanding of the effects of exposure to Cd on an organ system is important to overall human health.

#### 4.2.1. Renal Handling of Cadmium

Cd appears to gain access to renal epithelial cells via several different mechanisms. Cd ions have a strong affinity for sulfur groups and, thus, they may form complexes with select sulfhydryl (thiol)-containing biomolecules, such as GSH, cysteine (Cys) or homocysteine (Hcy) [95,96]. These Cd-thiol complexes may gain access to cells at the site of membrane transporters involved normally in the transport of endogenous amino acids, oligopeptides, organic anions, or organic cations (Table 2) [97,98]. One such example is the uptake of Cd via the organic cation transporter 2 (OCT2; *SLC22A2*) [99,100]. OCT2 is localized in the basolateral membrane of proximal tubular cells and is involved in the transport of a wide variety of cationic substances [101,102]. The exact species of Cd taken up by OCT2 has yet to be determined.

Cd may also be taken up by proximal tubules as a CdMT complex. Cd present in the liver becomes associated with MT to form CdMT [103,104], which may be released into sinusoidal blood following Cd-induced hepatocellular necrosis. CdMT is filtered freely at the glomerulus and is then thought to be taken up at the luminal plasma membrane of proximal tubular epithelial cells via receptor-mediated endocytosis [98,105,106,107,108,109,110,111,112,113]. This route of uptake appears to be a major route for entry of Cd at the luminal membrane of proximal tubular cells [114]. Indeed, the cells of the proximal convoluted tubule are the primary sites affected adversely by CdMT [103,107,109,115,116,117,118,119]. Following uptake by proximal tubular cells, CdMT is delivered to endosomes and lysosomes where Cd^2+^ is dissociated from MT and transported into the cytoplasm via the divalent metal transporter 1 (DMT1; *SLC11A2*) [106,120]. DMT1 is also localized in the luminal plasma membrane of the epithelial cells lining the ascending thick limb of the loop of Henle, the distal convoluted tubule, and the principal cells of the cortical collecting duct [121] where it may play a role in the uptake of Cd ions and lead to adverse effects within these cells.

Cd ions may also be taken up at the luminal membrane of proximal tubular cells via a mechanism involving ligand exchange. Under certain conditions, Cd may dissociate from MT or other ligands [122] and may be taken up subsequently by a cation transporter. Indeed, certain zinc transporters appear to be capable of utilizing Cd ions as substrates. In vitro studies in cultured renal epithelial cells have shown that ZIP8 (ZRT/IRT-like protein; *SLC39A8*) and ZIP14 (*SLC39A14*) are able to mediate the uptake of Cd ions [113,123,124,125,126]. Both of these carriers have been identified in the kidney [127] and appear to play important roles in the accumulation of Cd ions in proximal tubular cells. Interestingly, studies in HEK-293 cells have shown that a mutation (Ala391 to Thr391) in ZIP8 enhances Cd uptake into the kidney and vascular endothelial cells [128]. It is likely that Cd ions and Cd-thiol complexes utilize additional mechanisms to gain access to renal epithelial cells; however, those mechanisms and processes remain unclear at present.

Recent studies using HEK-293 cells stably transfected with multidrug and toxin extrusion protein (MATE1; *SLC47A1*) or MATE2-K (*SLC47A2*) have shown that these carriers are able to mediate the export of Cd from within proximal tubular cells into the tubular lumen [60]. MATE1 and MATE2-K are localized in the luminal membrane of proximal tubular cells [129,130] and appear to be involved in the urinary excretion of Cd. Other transporters localized in the luminal membrane that may play a role in the export of Cd include MRP2, MRP4, breast cancer resistance protein (BCRP; *ABCG2*), and P-glycoprotein. Studies in cultured human intestinal cells provide indirect evidence suggesting that MRP2 and P-glycoprotein may be involved in the transport of Cd [131]. Currently, there are no data supporting a role for MPR4 or BCRP in the transport of Cd.

#### 4.2.2. Renal Effects of Cadmium Exposure

Exposure to Cd is often assessed by measuring the concentration of Cd in urine and/or blood. In fact, urinary excretion of Cd is considered to be one of the most reliable indicators of renal and body burden of Cd. Detection of Cd in urine typically represents chronic levels of exposure [92,132,133] while plasma levels of Cd usually represent a more recent exposure, such as one occurring within the previous month [92,132,134]. Cd has a long half-life within the body, partly due to its incorporation into bone [87]. Therefore, following exposure, decades may be required for complete elimination of this toxic metal [92]. 

Following chronic exposure to Cd, approximately 50% of the total body stores accumulate in the kidney [135,136]. Thus, it is not surprising to find that this organ is one of the primary targets of Cd intoxication [137]. Renal accumulation of Cd leads to reduced GFR, polyuria, and generalized tubular dysfunction (i.e., Fanconi’s syndrome) [132,136,138]. One of the earliest signs of renal damage is the presence of urinary biomarkers such as kidney injury molecule-1 (Kim-1), β_2_-microglobulin, *N*-acetyl-β-d-glucosamidase (NAG), and cystatin C [132,137]. β_2_-microglobulin is a low molecular weight protein that is filtered freely at the glomerulus and is absorbed normally by proximal tubules [139]. Following tubular damage, a small fraction of β_2_-microglobulin escapes reabsorption and is excreted in urine. Alternatively, NAG is derived from mitochondria within proximal tubular epithelial cells and is released into the tubular fluid after cells are injured [140]. Kim-1, which is a transmembrane protein not normally detectable in urine, has also been shown to be a sensitive marker of renal tubular cell injury and/or death [141]. More recently, cystatin C, a cysteine protease inhibitor, has been shown to be a reliable biomarker of Cd-induced renal cell injury [137].

Recently, studies have shown that chronic exposure to even low levels of Cd can result in early signs of renal toxicity [138,142,143,144,145,146]. The earliest sign of Cd-induced renal damage is microproteinuria, which is usually characterized by the presence of β_2_-microglobulin in urine [88,92]. The observed microproteinuria may be due to a Cd-induced loss of megalin and cubilin, which mediate endocytosis of proteins along the proximal tubule [147,148,149,150]. Longer exposures to Cd cause shortening and loss of proximal tubular microvilli, which, consequently, leads to significant reductions in the number of transporters [147]. A reduced ability to absorb substrates taken up normally by proximal tubular cells can lead to the presence of a Fanconi’s syndrome characterized by glucosuria, aminoaciduria, hyperphosphaturia, and hypercalciuria [88,147]. In addition, autophagy and apoptosis are induced in intraglomerular mesangial cells [151,152], glomeruli are injured and, consequently, GFR is reduced [88,92,153,154]. The incidence of kidney stones also increases in individuals exposed chronically to (or to larger doses of) Cd, possibly due to the increased concentration of calcium in tubular fluid and urine [155]. Owing to the fact that the active form of vitamin D (1,25-dihydroxycholecalciferol) is formed in the kidneys, it is possible that renal injury would impede the conversion of the inactive form of this vitamin to the active form. Indeed, studies from Cd-polluted areas report an association between Cd-induced renal damage and lowered plasma levels of active vitamin D [156,157]. 

Exposure to Cd leads to increases in superoxide dismutase, catalase, glutathione peroxidase [158,159], and heme oxygenase [160]. Interestingly, a decrease in the expression of heme oxygenase leads to greater levels of Cd-induced apoptosis. Cd has also been shown to reduce the expression of certain miRNAs, specifically miRNA 125-a, and 125-b. Under normal conditions, miRNAs 125-a and 125-b are thought to be involved in the suppression of Cd-induced apoptosis [161]. Reductions in the expression of these two miRNAs would promote apoptosis induced by Cd and possibly other heavy metals.

Zinc has been shown to reduce renal toxicity induced by Cd [162,163,164], possibly by blocking the ability of Cd to alter anti-oxidant enzymes. Zinc also induces the renal and hepatic expression of MT [165]. Increased expression of MT will enhance the formation of MT-Cd complexes in the liver and kidney, which will enhance retention of a non-toxic form of Cd [166,167]. Zinc has also been shown to reduce Cd-induced apoptosis in renal cells [168] and may also compete with Cd for entry into cells at the site of select transporters [169].

Interestingly, Mg has also been shown to reduce Cd-induced toxicity. One possible pathway is via the stimulation of GSH synthesis, which would reduce the antioxidant effects of Cd [170,171]. In addition, Mg may compete with Cd at the site of transporters that mediate the uptake of Cd, thereby reducing the uptake of Cd [172]. Consequently, the presence of Mg reduces lipid peroxidation and oxidative stress [162,173]. This may be because Mg serves as a cofactor for enzymes needed to reduce ROS.

#### 4.2.3. CKD and the Effects of Cadmium Exposure on the Kidneys

Epidemiological studies have demonstrated a positive correlation between kidney disease and the renal accumulation of Cd in individuals exposed chronically to this metal [145,174,175,176,177,178]. Since a decline in the normal filtration capacity of the kidney is associated with CKD, exposure of individuals with CKD to Cd may potentiate the negative effects of disease-induced renal dysfunction. Consequently, it is reasonable to postulate that exposure of individuals with CKD to Cd may be especially detrimental to target organs. In diseased kidneys, the threshold at which nephrotoxic effects are observed may be lower than in healthy kidneys. Indeed, it has been suggested that long-term exposure to Cd exacerbates the CKD-related decline in GFR [179,180,181,182]. Moreover, exposure to Cd or other nephrotoxicants may further reduce or completely eliminate the renal functional reserve and the ability of the remaining functional renal mass to maintain normal homeostasis when challenged [12,183,184,185]. Indeed, Sprague-Dawley rats exposed chronically to oral Cd have been shown to have less renal functional reserve than unexposed rats [12]. Collectively, these studies suggest that exposure to Cd, and perhaps other nephrotoxicants, can abolish renal functional reserve, which may increase the susceptibility of these individuals to renal failure resulting from other risk factors such as hypertension and diabetes. 

Analyses of data from the 1999–2006 National Health and Nutrition Examination Surveys (NHANES) found that chronic exposure to low levels of Cd is associated with albuminuria. It can be postulated that continued exposure to Cd will enhance the risk of developing CKD [175]. Indeed, a cross-sectional study of individuals living in areas of Sri Lanka that are heavily contaminated with heavy metals, such as Cd, reported that exposure to Cd is a risk factor for the development of CKD [186]. Similarly, a cross-sectional study of individuals participating in the Korean National Health and Nutrition Examination Study (KHNANES) reported that exposure to Cd was associated with the development of CKD. This trend was particularly true in adults with hypertension and diabetes, which are considered to be major risk factors for CKD [187]. Despite multiple studies that appear to link exposure to Cd with the development of CKD, a few studies suggest that there is no connection between exposure to Cd and CKD. The discrepancy between these groups of studies suggests that additional environmental, dietary, and/or genetic factors may play a role in the susceptibility to Cd and the development of CKD [188,189].

Much of the data regarding the effects of Cd exposure on CKD comes from cross-sectional epidemiological studies because it is difficult to develop experimental models of chronic CKD. Alternatively, experimental reduction of renal mass via uninephrectomy creates a pathological scenario that is somewhat similar to that of CKD. In uninephrectomized animals, the remnant kidney undergoes significant compensatory changes in order to maintain normal fluid and solute homeostasis [9,190]. These changes include increased transcription and translation of numerous proteins, (including membrane transporters and metal-binding proteins (MT1 and MT2)) [9,190,191]. An increase in the number and/or activity of mechanisms involved in the proximal tubular uptake of Cd may enhance the nephropathy induced by this metal. When uninephrectomized and sham Sprague-Dawley rats were exposed to Cd, the renal burden of Cd was greater in the remnant kidney of uninephrectomized rats than in the corresponding kidney of sham rats [119]. In addition, the urinary excretion of NAG and Cd was greater in uninephrectomized rats than in sham rats. Furthermore, when a toxic dose of Cd was administered to each group of rats, it was found that uninephrectomized rats were more susceptible to the toxic effects of Cd than corresponding sham rats [119]. Considering these data, it is logical to propose that exposure to Cd following a reduction in functional renal mass may lead to more severe nephropathy [119]. 

Diseases such as hypertension and diabetes that affect renal health are common throughout the world. Therefore, it is important to understand the relationship between Cd exposure and superimposed diseases. Numerous epidemiological and animal studies have provided evidence suggesting an association between exposure to Cd and the occurrence and severity of diabetes [188,192,193,194]. While diabetes alone may lead to decreased GFR, albuminuria, and morphological alterations along the nephron [195], chronic exposure to Cd may enhance the onset of these negative renal effects [192]. Indeed, studies in which normal Sprague-Dawley rats were injected intraperitoneally with Cd showed that administration of Cd induced hyperglycemia [196,197]. This hyperglycemia may be the result of increases in levels of the gluconeogenic enzymes, glucose-6-phosphatase, fructose-1,6-diphosphatase, phosphoenol pyruvate carboxykinase, and pyruvate carboxylase [197]. Exposure of rats to Cd also appears to decrease the gene expression and release of insulin [198,199,200]. Taken together, these studies suggest that exposure to Cd may increase one’s susceptibility of developing diabetes. Exposure to Cd may also promote the development of signs and symptoms in a diabetic patient. Diabetes-induced renal pathology may be observed earlier in patients that are exposed chronically to low levels of Cd compared with un-exposed patients. This theory is supported by studies comparing streptozotocin-induced diabetic Wistar rats and non-diabetic Wistar rats [201]. The findings from these studies showed that urinary levels of protein, NAG, and γ-glutamyltransferase were greater in diabetic rats than in controls, suggesting that renal damage was more extensive in diabetic rats [201]. In addition, diabetic rats were found to excrete less Cd in urine and consequently had a greater renal burden of Cd than that of non-diabetic rats, suggesting a decrease in GFR [201]. In a similar study it was found that exposure of diabetic Sprague-Dawley rats to Cd significantly increased the urinary excretion of albumin, transferrin, and IgG [202]. In a separate study, varying concentrations of CdMT were injected into normal or obese hyperglycemic (ob/ob) mice [203]. Pathological signs of nephron damage (proteinuria and calciuria) were observed at lower concentrations of Cd in the ob/ob mice than in normal mice suggesting that the hyperglycemic state increases susceptibility to cadmium-induced nephropathy [203]. 

The results of multiple epidemiological studies correlate well with the aforementioned animal studies and provide additional support for the notion that exposure to Cd enhances the renal pathology associated with diabetes. In a cross-sectional study carried out in the Torres Strait Islands, located between Australia and New Guinea, investigators identified a strong positive correlation between urinary markers of Cd exposure and diabetic nephropathy [204]. Similarly, a cross-sectional study of 1699 Belgium subjects suggested that diabetic patients may be more susceptible to the nephrotoxic effects of Cd [205]. Moreover, Åkesson and colleagues assessed the effect of Cd exposure on diabetes-induced renal dysfunction in 10,766 subjects and reported that the nephrotoxic effects of Cd exposure could be observed at lower levels in diabetic patients compared with non-diabetic patients [138].

Interestingly, Cd levels in men and women appear to differ significantly. The body burden of Cd in women tends to be significantly greater than that in men. In a study of healthy Thai men and women, it was found that the average urinary excretion of Cd in non-smoking women was similar to that of men who smoked cigarettes [206]. In a study carried out in 57 non-smoking women, it was found that urinary and blood levels of cadmium correlated with age and body iron stores [207]. Women with lower serum ferritin were found to have higher levels of Cd [207,208]. In general, women have lower iron stores than men; when iron stores are low, the divalent metal transporter 1 (DMT1) in the intestine is upregulated to facilitate increased intestinal uptake of ferrous iron (Fe^2+^) [209]. DMT1 has also been shown to mediate the intestinal uptake of Cd [209,210]; therefore, upregulation of this carrier could potentially increase the absorption of dietary Cd ions from the lumen of the intestine. Indeed, it has been proposed that an increase in DMT1 expression and consequent increase in Cd absorption is the primary reason for the greater levels of Cd detected in women [206,207,208]. 

### 4.3. Lead 

Lead (Pb) is a toxic metal that usually exists bound to two or more other compounds. Pb is found throughout the environment, which is primarily due to various human activities. Pb compounds were found commonly in gasoline, batteries, pipes, and ammunition. In addition, Pb compounds were once used frequently as pigment in paints and ceramic glazes. When leaded gasoline was used as the primary source of fuel for automobiles, the environment became contaminated heavily with large quantities of Pb. In addition, the paint in many homes was Pb-based, containing up to 40% Pb [14]. Water pipes in older homes and water systems may contain Pb solder, which may leach into the water supply and lead to significant exposure to Pb compounds [211,212,213].

Occupational exposure to the inorganic form of Pb (Pb^2+^) can occur through welding processes, in the manufacture of Pb-containing batteries, lead smelting and refining, and in the production of pottery [14]. Of particular concern is the exposure of children to Pb^2+^, which occurs primarily via the ingestion of contaminated soil [14]. It is estimated that a large percentage of adults and children in the United States have blood lead levels that are higher than that which is considered to be safe [214,215,216].

Pb has been shown to have serious consequences on the nervous, circulatory, skeletal, renal, hematopoietic, and endocrine systems [14,217]. Pb poisoning is more common in children than adults are and is characterized by neurological symptoms such as headache, convulsions, ataxia, learning disorders, and hyperactive behavior [218]. Exposure to Pb may also result in nephropathy, renal adenocarcinoma, cardiovascular disease, and metabolic defects in bone [14,217,219]. 

#### 4.3.1. Renal Handling of Lead

Despite the nephropathy caused by exposure to Pb compounds, the mechanisms by which Pb enters target cells in the kidney are not well understood. Several mechanisms have been postulated to explain the transport of Pb^2+^ into and out of target cells (Table 3).

Endocytosis of Pb^2+^-protein complexes may serve as a route for the entry of this metal into cells [220]. Initially, a 63 KDa protein was identified as a Pb^2+^-binding protein (PbBP) in the cytosolic fraction of rat kidneys [221,222]. This protein was identified later as alpha-2-microglobulin [223,224]. Subsequent studies identified diazapine-binding inhibitor (DBI) and thymosin β-4 as additional PbBPs found in the kidney [220]. 

In addition, numerous in vivo and in vitro studies have described an interaction between Pb and Ca^2+^, suggesting that Pb^2+^ may utilize Ca^2+^ channels in order to gain entry into cells [225,226,227]. Additional studies suggest that the absorption of Pb^2+^ is inversely proportional to dietary levels of Ca^2+^ [228,229,230,231]. Indeed, an examination of approximately 3,000 children that were exposed to Pb^2+^ also revealed a relationship between dietary Ca^2+^ and blood levels of Pb [232]. It appears that low dietary intake of Ca^2+^ can lead to higher levels of Pb^2+^ in blood. The reverse of this relationship was also shown in that high intake of Ca^2+^ was associated with lower blood levels of Pb^2+^ [231,233,234]. Owing to the interaction between Pb^2+^ and Ca^2+^, it can be hypothesized that P^+^ enters cells through one or more Ca^2+^ channels. Moreover, since the atomic radius of Pb (1.81 Å) is smaller than that of Ca^2+^ (2.23 Å), it is possible that Pb may act as a mimic of Ca^2+^ at the site of Ca^2+^ transporters. 

Reabsorption of Ca^2+^ by proximal tubules is a two-step process with Ca^2+^ channels mediating the transport across the apical membrane and a Ca^2+^-ATPase on the basolateral membrane that mediates the movement of Ca^2+^ across the basolateral membrane [235]. Pb^2+^ appears to gain access to proximal tubular cells via Ca^2+^ channels on the apical membrane [236,237,238,239]. Studies in several different cell-types, including HEK293 cells, a human embryonic kidney cell-line, demonstrated that Pb^2+^ can enter cells via store-operated calcium channels (SOCs) [236,239,240]. It appears that Orai1 and stromal interacting protein 1 (STIM1), which are critical components of SOCs, play important roles in the cellular entry of Pb^2+^ [241]. Data from these studies also indicate that the flux of Pb through Ca^2+^ channels is a time- and concentration-dependent process and is approximately tenfold greater than that observed for Ca^2+^. Exit of Ca^2+^ at the basolateral membrane involves a Ca^2+^-ATPase. In vivo studies using erythrocytes indicate that Pb may substitute for Ca^2+^ at the site of the Ca^2+^-ATPase [237,242]. The ability of Pb to be transported by the Ca^2+^-ATPase in proximal tubular cells has not been shown; however, since the Ca^2+^-ATPase is homologous across cell-types, it is feasible to suggest that this transporter may mediate the movement of Pb across the basolateral membrane of proximal tubular cells.

Pb may also be transported out of at the apical membrane of proximal tubular cells into the lumen for eventual excretion in the urine. Experiments in rats suggest that Pb can be transported out of cells as a conjugate of GSH [243]. Though the transport of a GSH-Pb^2+^ complex was not demonstrated directly, it is possible that this complex may be a transportable form of Pb at the site of a transporter such as MRP2 or BCRP. These carriers are localized in the apical membrane of proximal tubular cells and mediate the export of a wide variety of compounds [52]; therefore, it is possible that MRP2 and BCRP may mediate the export of GSH-Pb complexes.

#### 4.3.2. Renal Effects of Lead Exposure

Owing to the role of the kidney in urinary excretion of toxicants, the kidney appears to be one of the primary sites of accumulation of Pb [244]. Exposure to low levels of Pb early in life have been shown to lead to glomerular hypertrophy, manifested specifically as an increase in the volume of glomerular capillaries [245]. Exposure to Pb may disrupt glomerular development which may result in renal insufficiency later in life. The tubules are also affected by exposure to Pb. Acute exposure can lead to generalized defects in solute and amino acid transport in renal tubules, leading to a Fanconi syndrome [225,246,247]. Chronic exposure to Pb may lead to progressive tubulointerstitial nephritis that is characterized by infiltration of leukocytes, interstitial fibrosis, and tubular atrophy [248]. Similarly, when Wistar rats were exposed to lead acetate, tubular degeneration, intraluminal hyaline casts, blood vessel congestion, perivascular fibrosis, and vascular edema were observed [249]. Since Pb tends to induce injury in proximal tubules, kidney injury molecule-1 (KIM1) [250] and alpha-glutathione *S*-transferase (αGST) [251] appear to be appropriate urinary biomarkers for Pb-induced renal injury.

One of the primary cellular effects of exposure to Pb is the induction of oxidative stress in the cells of the kidney [252,253]. Exposure of mice to lead acetate enhances the production of reactive oxygen species and reduces the mRNA expression of enzymes necessary to counteract oxidative stress (i.e., catalase, superoxide dismutase, glutathione *S*-transferase, glutathione peroxidase). Alternatively, exposure of mice to Pb enhanced mRNA expression of transforming growth factor-β1 (TGFβ1), monocyte chemoattractant protein-1 (MCP-1), and alpha-2 macroglobulin (α-2M), which lead to inflammatory processes [249]. Exposure to Pb has also been shown to lead to lipid oxidation and DNA fragmentation [254]. 

It appears that mitochondria play an important role in Pb-mediated injury. Oxidative stress within the cell may lead to alterations in the regulation of the mitochondria permeability transition pore (MPTP) [255], which normally mediates the osmotic influx of water into the mitochondrial matrix [256]. Exposure of cells to Pb has been shown to induce abnormal opening of the MPTP which leads to swelling of mitochondria, changes in membrane potential, and initiation of apoptosis [253,256,257,258]. Exposure of cells to Pb also leads to structural alterations in the mitochondria such as distortion of the mitochondrial cristae and swelling and rupture of the outer membrane [255]. 

Pb, like the cationic species of some of the other toxic metals, is also capable of acting as a functional mimic of endogenous ions at intracellular binding sites. It has been shown to be a functional substitute for Ca^2+^ at the site of calmodulin [259,260], a protein that plays a role in the regulation of intracellular Ca^2+^ [261]. In addition, the activity of protein kinase C (PKC) may be affected by the binding of Pb. Under normal conditions, Ca^2+^ activates PKC, which mediates numerous intracellular processes and signaling cascades. Interestingly, it has been reported that Pb is a better activator of protein kinase C than is Ca^2+^ [227]. It is important to note that the actions of intracellular Ca^2+^, unlike Pb, are highly regulated. Therefore, the binding of Pb^2+^ to an enzyme such as protein kinase C may activate the enzyme unnecessarily and result in deleterious effects. Pb may also act as a mimic of Ca^2+^ at binding sites of cellular junctions. Many junctional complexes require Ca^2+^ in order to maintain their integrity and, thus, binding of Pb instead of Ca^2+^ may compromise the integrity of the junctional complex. 

In addition, exposure to Pb has been shown to alter the subcellular distribution of calcium in renal cells [262]. Specifically, exposure to Pb resulted in elevated levels of calcium in the cytoplasm and mitochondria while calcium levels in the endoplasmic reticulum were depleted. These changes in intracellular calcium ratios may lead to significant alterations in intracellular signaling pathways and eventual apoptosis [262]. Interestingly, administration of the calcium channel blockers, verapamil and nimodipine, have been shown to decrease lipid oxidation and increase the activities of superoxide dismutase and glutathione peroxidase [263]. 

#### 4.3.3. CKD and the Effects of Lead Exposure on the Kidneys

Although it was once thought that exposure to Pb was not directly associated with renal disease [264], it appears that there may be a direct relationship between exposure to Pb and the development of kidney disease [265,266,267]. A study of industrial workers exposed occupationally to Pb did not detect overt signs of renal dysfunction. However, analyses of biochemical markers in urine suggested that Pb induces changes at the cellular level even if renal function is not compromised [248]. Not surprisingly, individuals with higher blood Pb concentrations have a higher risk of renal injury [268]. A cross-sectional study of individuals in Mexico suggested that serum creatinine levels correlate positively with blood Pb [269]. This finding provides some indication that exposure to Pb may lead to a reduction in GFR. Similarly, a cross-sectional study of Korean adults demonstrated a positive correlation between blood Pb levels and renal dysfunction [270]. Additionally, erythrocyte Pb appears to be associated with renal injury [271].

### 4.4. Mercury

Mercury is a toxic metal found in many environmental and industrial settings. It exists in elemental (metallic), inorganic, and/or organic forms. Elemental mercury (Hg^0^) is unique in that it exists as a liquid at room temperature. Inorganic mercury may be found as mercurous (Hg^1+^) or mercuric (Hg^2+^) ions, which are usually bound to chlorine, sulfur, or oxygen to form mercurous or mercuric salts. There are several common forms of organic mercury such as phenylmercury, dimethylmercury, and monomethylmercury. Of these forms, methylmercury (CH_3_Hg^+^) is encountered most frequently in the environment. It is formed predominantly when inorganic mercuric ions are methylated by microorganisms present in soil and water [272,273,274,275]. 

Humans are exposed to mercuric compounds via occupational, environmental, and dietary sources [272,273,275,276]. The majority of human exposure is due to the ingestion of food contaminated with CH_3_Hg^+^. Upon ingestion, CH_3_Hg^+^ is absorbed readily by the gastrointestinal tract of humans and other mammals [272]. Mercuric ions can then enter systemic circulation where they can be delivered to target organs. It is important to note that approximately 14 days after exposure to CH_3_Hg^+^, a fraction of absorbed CH_3_Hg^+^ may be oxidized to form Hg^2+^ [277,278,279,280,281]. 

#### 4.4.1. Renal Handling of Mercury

Inorganic and organic forms of mercury accumulate readily in the kidney. The kidney is the primary site of accumulation of and intoxication by inorganic forms of mercury. The accumulation of Hg^2+^ in renal tubular cells, primarily those of the proximal tubule, occurs rapidly with approximately 50% of a nontoxic dose found in the kidneys after a few hours of exposure [275,282]. Organic forms of mercury, which primarily affect the central nervous system, may also have serious toxicological effects in the kidney [283,284,285,286]. It is important to note that within biological systems, mercurous, mercuric, or methylmercuric ions do not exist as inorganic salts, or in an unbound, “free” ionic state [287]. Rather, mercuric ions are bound to one or more thiol-containing biomolecules, such as GSH, Cys, Hcy, *N*-acetylcysteine (NAC), and albumin. For Hg^2+^, this bonding occurs in a linear II, coordinate covalent manner while thiol-conjugates of CH_3_Hg^+^ form linear I, coordinate covalent complexes [288,289].

At the cellular level, mercuric ions appear to gain access to proximal tubular cells via mechanisms present in the luminal and basolateral plasma membranes (Table 4) [290,291,292,293,294]. In vitro studies utilizing isolated perfused proximal tubules suggest that a Cys *S*-conjugate of Hg^2+^ (Cys-*S*-Hg-*S*-Cys) is the primary form of Hg^2+^ transported across the luminal membrane of proximal tubular cells [295,296,297]. Additional studies indicate that amino acid transporters located in the luminal plasma membrane are likely involved in the uptake of mercuric conjugates from the tubular lumen [290,291]. It has been hypothesized that since Cys-*S*-Hg-*S*-Cys is similar in size and shape to the amino acid cystine, this mercuric conjugate may be a substrate of a cystine transporter. Studies using Madin-Darby Canine Kidney (MDCK) cells transfected with the sodium-independent cystine transporter, system b^0,+^, provide strong evidence implicating this carrier in the cellular uptake of Cys-*S*-Hg-*S*-Cys [298]. Similar studies have also identified the Hcy *S*-conjugate of Hg (Hcy-*S*-Hg-*S*-Hcy) as a substrate for system b^0,+^ [299]. Furthermore, evidence from studies in *Xenopus laevis* oocytes suggests that Cys- and Hcy-*S*-conjugates of CH_3_Hg^+^ (Cys-*S*-CH_3_Hg^+^ and Hcy-*S*-CH_3_Hg^+^, respectively) are substrates of the sodium-dependent amino acid carrier, system B^0,+^ [300]. Currently, there are no data supporting a role for system B^0,+^ in the uptake of Cys-*S*-Hg-*S*-Cys or Hcy-*S*-Hg-*S*-Hcy [300].

Approximately 40–60% of the mercury that accumulates in proximal tubular cells is taken up at the basolateral plasma membrane [292,294,297,301,302,303,304]. Numerous in vitro studies using cultured MDCK cells stably transfected with the organic anion transporter, OAT1 (*SLC22A6*), provide strong evidence indicating that mercuric conjugates of Cys, Hcy, and NAC (NAC-*S*-Hg-*S*-NAC) are taken up by this carrier [305,306,307,308,309,310,311,312,313,314,315]. Cys-*S*-Hg-*S*-Cys has also been shown to be a transportable substrate of OAT3 [316]. Both, OAT1 and OAT3 (*SLC22A8*) are localized in the basolateral plasma membrane of proximal tubular epithelial cells [307,308]. Based on current scientific knowledge, it appears that OAT1 is the primary mechanism involved in the basolateral transport of Cys-*S*-Hg-*S*-Cys, NAC-*S*-Hg-*S*-NAC, and Hcy-*S*-Hg-*S*-Hcy into proximal tubular cells [292,293,297,301,302,303,304,316,317,318]. In addition to conjugates of Hg^2+^, Cys-, NAC-, and Hcy-*S*-conjugates of CH_3_Hg^+^ have also been identified as substrates for OAT1 [313,314,315]. Collectively, these data provide strong support for a role of OAT1 and OAT3 in the basolateral uptake of certain mercuric complexes.

Once mercuric ions gain access to the intracellular compartment of cells, they form strong bonds with protein and non-protein thiol-containing biomolecules. Intracellular mercuric ions also stimulate, and bind to, MT and/or GSH [319,320]. Binding to these biomolecules often prevents or reduces greatly the export of mercuric ions from the cell. It is well-documented that mercuric ions can be extracted from renal tubular cells following treatment with a metal chelating agent, such as 2,3-bis(sulfanyl)propane-1-sulfonic acid (formally known as 2,3-dimercaptopropane-1-sulfonic acid; DMPS) [321] or 2,3-dimercaptosuccinic acid (DMSA) [321,322,323,324,325,326,327]. It appears that DMPS and DMSA gain access to proximal tubular cells at the basolateral membrane via OAT1, OAT3 and/or the sodium-dependent dicarboxylate transporter (NaC2) [328,329,330,331]. Once inside the cell, it is hypothesized that DMPS and DMSA form complexes with intracellular Hg^2+^ and/or CH_3_Hg^+^ and that these complexes are then exported across the luminal membrane via MRP2 or BCRP [332,333,334,335]. Interestingly, NAC also appears to mediate the extraction of mercuric ions following exposure of rats to CH_3_Hg^+^ [336,337]. Collectively, these data provide solid evidence for the hypothesis that MRP2 plays an important role in the renal elimination of mercuric ions following exposure to forms of Hg^+2^ or CH_3_Hg^+^. 

#### 4.4.2. Renal Effects of Mercury Exposure

Exposure to all forms of mercury can have nephrotoxic effects [283,284,285,286]; however exposure to conjugates of Hg^2+^ leads to the most severe nephropathy. The *pars recta* of the proximal tubule appears to be the most sensitive to the toxic effects of mercury and is usually the first segment of the nephron affected by exposure to mercuric compounds [275]. The *pars convoluta* and distal segments of the nephron are not usually affected by exposure to low doses of mercury, but exposure to higher doses can lead to injury and necrosis in these segments [338,339,340]. Electron microscopic analyses of Hg-induced toxicological changes documented early alterations in mitochondrial structure and the presence of pyknotic nuclei. Approximately six hours after exposure, cells begin to lose microvilli, mitochondrial swelling worsens, and dilation of the endoplasmic reticulum can be detected [338]. Reductions in enzymatic activity in the *pars recta* have also been described [339]. Twelve hours after exposure to HgCl_2_, electron microscopic analyses of cells revealed rupture of the plasma membrane, loss of microvilli, decreased contact with the basement membrane, and destruction of cell shape [341]. After 24 h, cellular fragments can be identified in the tubular lumen, junctional complexes between cells are absent, and nuclear structure is compromised [339,340,341]. When tubular epithelial cells are injured and die, numerous brush-border and intracellular enzymes, such as alkaline phosphatase, γ-glutamyltransferase, lactate dehydrogenase, aspartate aminotransferase (AST), and alanine aminotransferase (ALT), can be identified in urine [325,342,343,344,345,346]. As the extent of mercury-induced renal injury progresses, there is also a simultaneous increase in the urinary excretion of mercuric species [347,348,349]. 

Exposure to HgCl_2_ can also have detrimental effects on glomeruli. Chronic exposure of rats to a non-nephrotoxic dose of HgCl_2_ led to tubular, interstitial, and glomerular lesions [350]. Similarly, in rats exposed chronically to methylmercury, fibrotic changes were observed in glomeruli, and deposits of IgG, IgM, and C3 were detected along the glomerular basement membrane [351]. Furthermore, an analysis of data from patients exposed chronically to mercuric compounds indicates that exposure to mercury can lead to glomerular injury and disease [352]. Collectively, these studies suggest that chronic exposure to Hg^2+^ or CH_3_Hg^+^ may lead to the development of membranous glomerulonephritis. Glomerular alterations such as fibrosis and glomerulonephritis often lead to reductions in GFR. Thus, it is not surprising that reductions in GFR have been observed after exposure to mercuric compounds [338,353].

#### 4.4.3. CKD and the Effects of Mercury Exposure on the Kidney

Although individuals with CKD make up a significant percentage of the population, and exposure to mercury and mercuric compounds is an important environmental problem, the information about the relationship between diseased kidneys and the effects of mercury exposure remains sparse. Considering the lack of epidemiological studies related to the exposure of patients with CKD to mercury, the current review will focus on experimental studies using models of reduced renal mass and CKD. 

There is not an animal model that perfectly mimics the renal and systemic changes that occur as part of CKD. However, the decline in functional renal mass that occurs in patients with CKD is similar to an experimental model in which animals are 50% or 75% nephrectomized. Although the nephrectomized rat models are not exact mimics of CKD, similarities exist between these systems. In each, a significant number of nephrons are lost and remaining nephrons must go through a compensatory, hypertrophic phase in order to maintain normal fluid and solute homeostasis [3,9,190]. Hyperfiltration, hyperperfusion, and an increase in the levels of certain proteins occur in hypertrophied nephrons. These changes may lead to these nephrons being exposed to higher levels of nephrotoxicants such as mercury, which may enhance the susceptibility of these nephrons to the harmful effects of mercury or other nephrotoxicants [345,354,355,356,357,358,359,360,361].

Studies using 50% nephrectomized rats as models of the early stages of CKD indicate that acute renal failure was more pronounced in nephrectomized rats exposed to a nephrotoxic dose of HgCl_2_ than in corresponding sham rats [345,354,356]. Exposure to HgCl_2_ led to glomerular and tubular dysfunction, which appeared to be more severe in 50% nephrectomized rats than in sham rats [354]. It was found that mercury-induced proximal tubular necrosis was more extensive in 50% nephrectomized animals than in sham animals. Additionally, the urinary excretion of cellular enzymes and plasma proteins, including lactate dehydrogenase, γ-glutamyltransferase and albumin, was greater in 50% nephrectomized animals than in sham animals [13,345]. 

**Figure 2:**
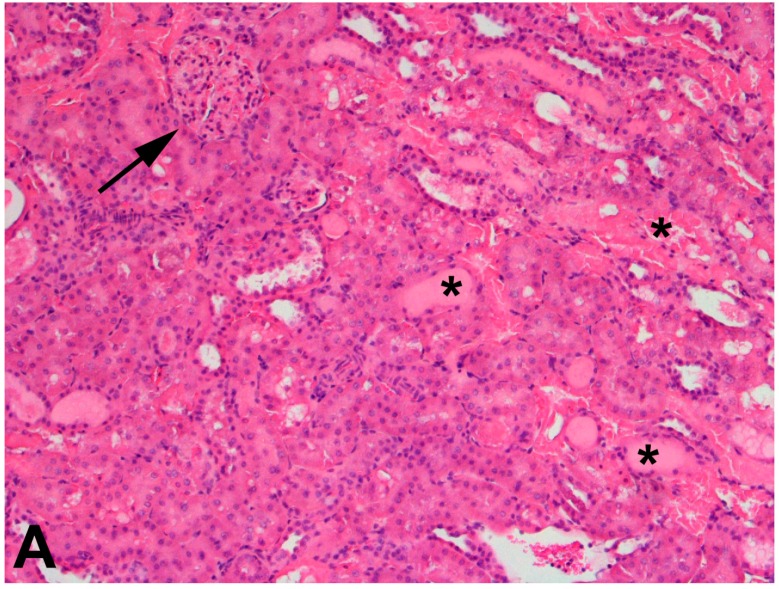

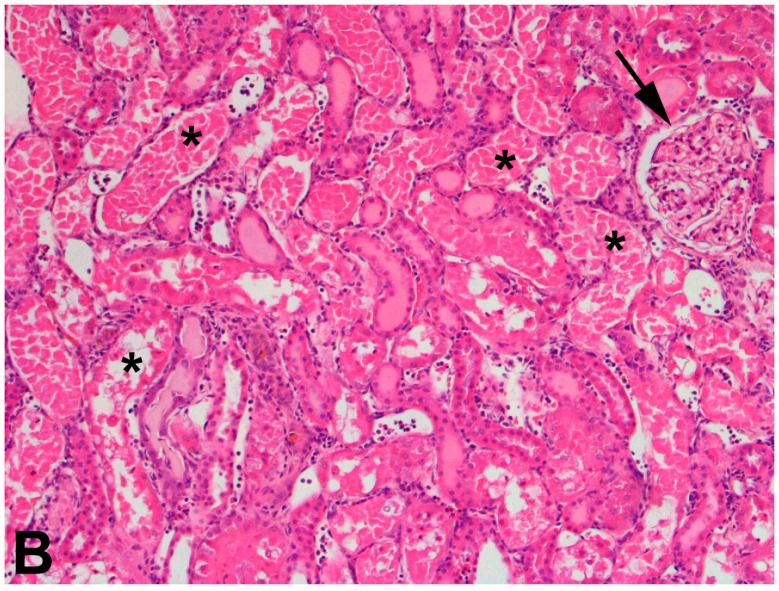
Histological sections of kidneys from Sham or 75% nephrectomized Wistar rats exposed to 2.5 μmol kg^−1^ HgCl_2_. Panel (**A**) shows a representative section of kidney from Sham rats exposed to2.5 μmol kg^−1^ HgCl_2_. This section displays a normal glomerulus (arrow) and a mix of normal and injured tubules (*). In addition, there is an infiltration of lymphocytes, which is likely one of the first responses to the inflammation caused by exposure to mercury. Panel (**B**) shows a representative section of kidney from 75% nephrectomized rats exposed to 2.5 μmol kg^−1^ HgCl_2_. Glomeruli (arrows) were hypertrophied and tubular necrosis (*) was widespread. The degree of injury in the 75% nephrectomized rats was significantly greater than that in corresponding Sham rats, suggesting that rats with reduced renal mass may be more sensitive to the nephrotoxicants such as mercury, than rats with normal renal mass. Magnification, 100×.

The use of 75% nephrectomized rats as models of CKD facilitates the study of a later stage of disease. The handling of mercury in these rats is significantly different than that in 50% nephrectomized or sham rats. Since the GFR of these rats is reduced significantly, total renal accumulation of mercury is significantly lower than in 50% nephrectomized or sham rats. However, the accumulation of mercury per g kidney is significantly greater in 75% nephrectomized rats than in shams, suggesting that the mercury uptake into individual cells may be greater in the remnant renal mass from 75% nephrectomized animals than in kidneys of sham animals [362]. It is also notable that the expression of mRNA encoding OAT1 and MRP2 also increased in the remnant renal mass of 75% nephrectomized rats. The enhanced expression of these carriers may be the underlying cause of the increased uptake of mercury into renal cells of the 75% nephrectomized rat [362]. Indeed, exposure of 75% nephrectomized rats to HgCl_2_ leads to renal injury that is more extensive and severe than that in corresponding sham rats (Figure 2). 

Collectively, the results of these studies suggest that kidneys of animals with reduced renal mass are more susceptible to the toxic effects of mercury. Similarly, individuals who have reduced renal function, due to CKD or other disease processes, may be more susceptible to renal injury following exposure to a nephrotoxicant such as mercury.

## 5. Summary

CKD has been well-studied and the structural and functional changes that are associated with it have been characterized extensively. However, there is little information regarding the response of injured kidneys to environmental toxicants such as arsenic, cadmium, lead, or mercury. Because of the prevalence of these metals in the environment, human exposure to one or more of these metals is nearly unavoidable. Furthermore, it is well-known that acute and chronic exposures to one or a combination of these toxic metals can be detrimental to the kidneys of normal adults. Therefore, it can be postulated that exposure of CKD patients to these metals may lead to additional reductions in renal function. Individuals with compromised renal function are especially susceptible to nephrotoxicants. The few studies available have demonstrated an association between exposure to heavy metals and an increase in the incidence and severity of renal disease. It is important to note that early signs of renal dysfunction often go unnoticed, thus, individuals with reduced renal function are often unaware that they are at risk during the early stages of disease [363,364]. Exposure to one or more nephrotoxicants (such as heavy metals) may occur during this early period and this exposure may be especially detrimental to these individuals in that it may enhance morbidity and/or mortality. Therefore, a thorough and complete understanding of the way in which nephrotoxicants are handled by dysfunctional kidneys is of utmost importance. Because of the paucity of data available on this topic, additional studies are clearly necessary. 

## Figures and Tables

**Table 1:** Potential mechanisms involved in the renal tubular transport of arsenic.

	Transport Mechanism	Species
Apical uptake	?	?
Basolateral uptake	GLUT1	As^III^ and MAs^III^
	GLUT5	As^III^ and MAs^III^
	Aquaporin 3	As^III^
	OATP2B1	?
Apical export	MRP2	As(GS)_3_, MAs(GS)_2_
	P-glycoprotein	iAs, As(GS)_3_, MAs(GS)_2_
	MRP4	iAs^III^, iAs^V^, MAs^III^, MAs^V^, DMA^V^
	MATE	?
Basolateral export	GLUT1	As^III^ and MAs^III^
	GLUT5	As^III^ and MAs^III^
	OATP2B1	As^III^ and MAs^III^

GLUT: glucose transporter; OATP: organic anion transporting polypeptide; MRP: multidrug resistance-associated protein; MATE: metal and toxicant extrusion protein.

**Table 2:** Potential mechanisms involved in the renal tubular transport of cadmium.

	Transport Mechanism	Species
Apical uptake	Receptor-mediated endocytosis	CdMT
	DMT1	Cd^2+^
	ZIP8	Cd^2+^
	ZIP14	Cd^2+^
Basolateral uptake	OCT2	Thiol *S*-conjugate of Cd
Apical export	MATE1	Cd^2+^
	MATE2-K	Cd^2+^
	MRP2	Thiol *S*-conjugate of Cd
	P-glycoprotein	Thiol *S*-conjugate of Cd
	MRP4	?
	BCRP	?
Basolateral export	?	?

DMT: divalent metal transporter; ZIP: ZRT/IRT-like protein; MATE: metal and toxicant extrusion protein; MRP: multidrug resistance-associated protein; BCRP: breast cancer resistance protein.

**Table 3:** Potential mechanisms involved in the renal tubular transport of lead.

	Transport Mechanism	Species
Apical uptake	Receptor-mediated endocytosis	Pb-protein complexes
	Ca^2+^ channels	Pb^2+^
Basolateral uptake	?	?
Apical export	MRP2	GSH-Pb
	BCRP	GSH-Pb
Basolateral export	Ca^2+^-ATPase	Pb^2+^

MRP: multidrug resistance-associated protein; BCRP: breast cancer resistance protein.

**Table 4:** Potential mechanisms involved in the renal tubular transport of mercury.

	Transport Mechanism	Species
Apical uptake	System b^0,+^	Cys- and Hcy-*S*-conjugates of Hg^2+^
	System B^0,+^	Cys-*S*-conjugates of MeHg
Basolateral uptake	OAT1	Cys-, Hcy-, NAC-*S*-conjugates of Hg^2+^ and MeHg
	OAT3	Cys-*S*-conjugates of Hg^2+^
Apical export	MRP2	DMPS-, DMSA-, NAC-*S*-conjugates of Hg^2+^ DMPS-, DMSA-*S*-conjugates of MeHg
	BCRP	DMPS- and Cys-*S*-conjugates of Hg^2+^
Basolateral export	?	?

OAT: organic anion transporter; MRP: multidrug resistance-associated protein; BCRP: breast cancer resistance protein.
